# Engineering electrode interfaces for telecom-band photodetection in MoS_2_/Au heterostructures via sub-band light absorption

**DOI:** 10.1038/s41377-023-01308-x

**Published:** 2023-11-23

**Authors:** Chengyun Hong, Saejin Oh, Vu Khac Dat, Sangyeon Pak, SeungNam Cha, Kyung-Hun Ko, Gyung-Min Choi, Tony Low, Sang-Hyun Oh, Ji-Hee Kim

**Affiliations:** 1 Department of Energy Science, Sungkyunkwan University, Suwon, 16419 Republic of Korea; 2 Center for Integrated Nanostructure Physics (CINAP), Institute for Basic Science (IBS), Sungkyunkwan University, Suwon, 16419 Republic of Korea; 3 School of Electronic and Electrical Engineering, Hongik University, Seoul, 04066 Republic of Korea; 4 Department of Physics, Sungkyunkwan University, Suwon, 16419 Republic of Korea; 5 Department of Electrical and Computer Engineering, University of Minnesota, Minneapolis, MN 55455 USA; 6 Department of Physics, Pusan National University, Busan, 46241 Republic of Korea

**Keywords:** Electronics, photonics and device physics, Supercontinuum generation

## Abstract

Transition metal dichalcogenide (TMD) layered semiconductors possess immense potential in the design of photonic, electronic, optoelectronic, and sensor devices. However, the sub-bandgap light absorption of TMD in the range from near-infrared (NIR) to short-wavelength infrared (SWIR) is insufficient for applications beyond the bandgap limit. Herein, we report that the sub-bandgap photoresponse of MoS_2_/Au heterostructures can be robustly modulated by the electrode fabrication method employed. We observed up to 60% sub-bandgap absorption in the MoS_2_/Au heterostructure, which includes the hybridized interface, where the Au layer was applied via sputter deposition. The greatly enhanced absorption of sub-bandgap light is due to the planar cavity formed by MoS_2_ and Au; as such, the absorption spectrum can be tuned by altering the thickness of the MoS_2_ layer. Photocurrent in the SWIR wavelength range increases due to increased absorption, which means that broad wavelength detection from visible toward SWIR is possible. We also achieved rapid photoresponse (~150 µs) and high responsivity (17 mA W^−1^) at an excitation wavelength of 1550 nm. Our findings demonstrate a facile method for optical property modulation using metal electrode engineering and for realizing SWIR photodetection in wide-bandgap 2D materials.

## Abstract (web summary)

Near-infrared light absorption in 2D materials can be engineered using electrode fabrication methods, which were exploited to construct sensitive telecom-band photodetectors based on MoS_2_.

## Graphical abstract

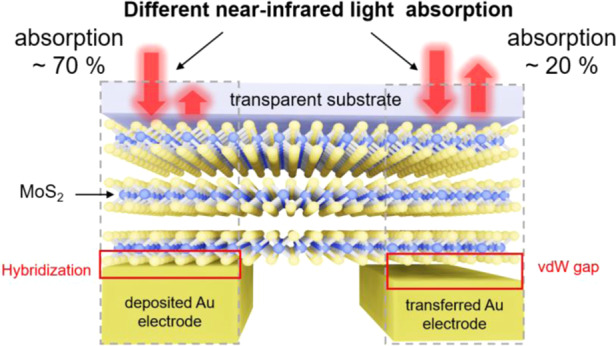

## Introduction

Transition metal dichalcogenides (TMDs) have attracted considerable attention as flexible and highly sensitive photodetectors due to their exceptional electrical, optical, and mechanical properties^1,2^. Among them, molybdenum disulfide (MoS_2_) is a promising candidate for post-silicon semiconductor materials owing to its outstanding chemical stability, high carrier mobility, and robust light-matter interaction. Its exotic physical properties and potential for innovative uses have been extensively studied over the past decade^3,4^. Moreover, significant progress has been achieved in developing stable and large-area thin film growth of semiconducting 2H-phase and active handling methods^5–7^. Benefit from those efforts from material and device engineering, various results have been reported for achieving high-performance photodetectors based on MoS_2_, such as high photodetection responsivity up to ~10^6^ A W^−1^^8^ or ultrafast photoresponse time of 3 ps^9^ in the visible wavelength range. However, the negligible absorption in the near-infrared regime impedes its application on the infrared photodetector. Although the multilayer TMD has a relatively small indirect band gap of 1.3 eV, the absorption below the optical band is insignificant, and the photocurrent decreases dramatically as well^10^. Though there have various small bandgap 2D materials like black phosphorous^11^, MoTe_2_^12^, Bi_2_O_2_Se^13^, PdSe_2_^14^, and PtSe_2_^15^ have been reported for infrared photodetectors, these materials encounter challenges in practical applications due to low stability, large dark current, limited growth techniques, and reliance on the novel and rare-earth materials. Thus, employing stable 2D materials with high band gaps holds promise in achieving robust and practical near-infrared photodetection capabilities.

There are several approaches that can realize the sub-bandgap photodetection with large bandgap materials like MoS_2_: (I) utilizing the sub-bandgap adoption of highly defective samples^16–18^, (II) utilizing the photothermal effects^19^, or (III) utilizing the hot electrons generated by the metal nanostructure or metal electrodes^20,21^. However, significant disadvantages exist in those mechanisms. MoS_2_-based photodetector adopting highly defective samples or utilizing the photothermal effect demonstrate low photodetection response to several hundred milliseconds or even slower^22,23^, due to relatively slow processes of defect-trapped carrier dynamics and thermal-assisted photoconductivity changes. Photodetection based on hot electron transfer^24–26^ from the metal to the semiconductor is an alternative way to realize sub-bandgap photodetection without sacrificing response speed. However, the hot electron photodetectors based on MoS_2_ at 1550 nm is only limited on the application of waveguide integrated system because hot carrier transfer efficiency depends strongly on the energy of light, resulting in negligible photocurrent in metal-semiconductor-metal (MSM) lateral device structure. As such, despite significant interest in expanding photodetection to the sub-bandgap regime, challenges such as low response speed and poor responsivities have hindered progress in this area. In the short wavelength infrared (SWIR) up to 1550 nm, there is an imperative need to develop new approaches that can simultaneously provide both high responsivity and fast response speed.

Metal contact is an essential part of modern electronic and optoelectronic devices and has attracted great interest in 2D material research^27–30^. Metal contact not only affects device performance electrically via the Schottky junction^31^, but can also impact performance as a consequence of changing optical properties. By reducing the lateral size of an electrode to the sub-micron scale to match the plasmon frequency, one can achieve increased sub-bandgap absorption, resulting in higher photoresponsivity in the sub-bandgap regime due to hot electron transfer^32^. Moreover, by modulating the 2D material thickness to match the Fabry-Pérot(F-P)-like cavity resonance in TMD/Ag heterostructure, it becomes possible to realize a considerable increase in absorption and external quantum efficiency^33,34^. However, the interface condition-dependent optical properties of 2D materials are not well-studied, and these may be strongly dependent on the electrode fabrication method. The interface between the deposited metal and 2D material is highly hybridized with many defect sites^35,36^, which can affect the optical properties of 2D material in the sub-bandgap regime^37^. Therefore, the condition of this interface offers an additional knob to control the optical properties of the 2D heterostructure and may thus provide a facile mechanism for sub-bandgap light detection.

Here, we discovered a universal approach to realize the telecom-band photodetection with large band gap TMD materials via electrode engineering. Combining the sub-bandgap absorption in the hybridized TMD/Au interface and the F-P-like cavity induced sub-bandgap absorption increase, high IR absorption of up to 60% can be realized by using industrial metal electrode deposition methods, with strong IR light absorption observed over a broad wavelength range of 800 ~ 1600 nm. The thickness of the MoS_2_ layer is critical because it determines the resonance frequency of the F–P-like cavity formed in the MoS_2_-Au heterostructure. We studied the NIR light-induced photocurrent generation mechanism using photocurrent mapping and determined that a MoS_2_ layer fabricated onto an Au electrode with the deposition method exhibited markedly higher photocurrent than transferred electrodes due to the strong IR absorption. Wavelengths of 1310 nm and 1550 nm can be detected when the light illuminates the MoS_2_/sputter-deposited Au (d-Au) electrode, with photoresponsivity of 35 mA W^−1^ at 1310 nm and 17 mA W^−1^ at 1550 nm and a fast response rate of 140 ~ 150 μs due to increased light absorption in the heterostructure. Our results highlight the critical role of interfacial engineering on optical absorption and photocurrent generation and provided new approach for high performance sub-bandgap photodetection with large band gap 2D materials.

## Results

### Sub-bandgap light absorption in MoS_2_ via engineering of the electrode interface

**Fig. 1:**
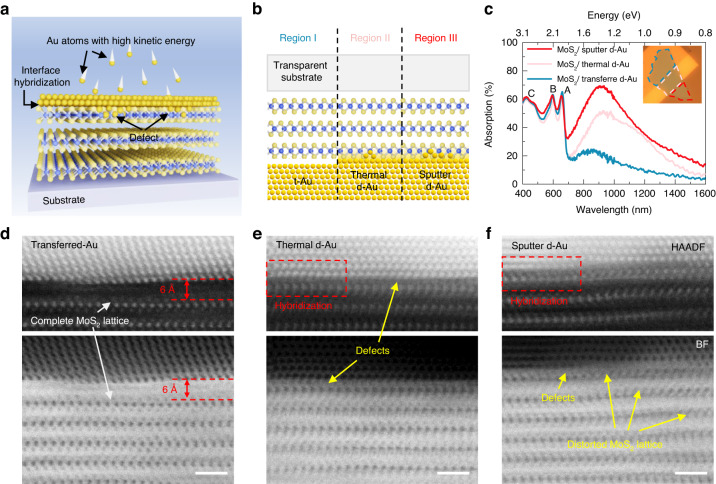
Robust sub-bandgap light absorption in MoS_2_ with different Au interface conditions. **a** Schematic of the Au deposition process, demonstrating how Au atoms with high kinetic energy can penetrate the MoS_2_ lattice and form a hybridized interlayer at the MoS_2_/Au interface. **b** Schematic of transparent electrode/MoS_2_/Au samples in which Region I of the Au film is formed by transferred Au, Region II is thermal deposition, and Region III is formed by sputter deposition. **c** Absorption spectra of MoS_2_ on the three different Au interfaces depicted in panel b. Inset shows optical micrograph of the measured sample, with the three different Au interfaces marked by dashed lines. The different colours in three different Au in the OM image are from different Au/PMMA interface, due to the instability of the PMMA. The thickness of MoS_2_ used in here is 39 nm as shown in Fig. S20. **d**–**f** Scanning transmission electron microscopy (STEM) image of the cross-section in the three different MoS_2_/Au interfaces. Top panels show high-angle annular dark field (HAADF) images and bottom panels show brightfield (BF) images. The defects in (**e**), (**f**) are pointing the S vacancies in the interfaces. Scale bar: 1 nm

**Fig. 2:**
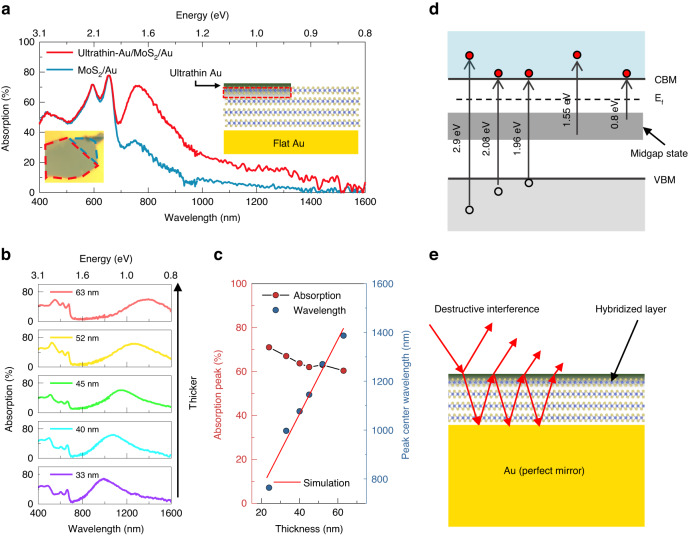
Modulation of absorption spectra in a Fabry–Pérot (F–P) cavity with MoS_2_ layers of varying thickness. **a** Absorption spectra of MoS_2_/Au heterostructures with (red) and without (blue) a top layer of ultrathin (~2.3 nm) Au applied via the sputter method (schematic shown in lower inset). Upper right inset shows optical micrograph of the measured sample, with coloured dashed lines indicating the two sectors being analysed. The thickness of the MoS_2_ is 24 nm, and it was also included in the analysis of (**c**). **b** Absorption spectra show a redshift of the NIR absorption peak as the thickness of the MoS_2_ layer increases. **c** Absorption peak value (left *y*-axis) and the center of the peak wavelength (right *y*-axis) for the same six samples shown in panel (**b**). Simulation data (red line) were obtained using the transfer matrix method, demonstrating the formation of an F-P-like cavity in the MoS_2_/Au heterostructure. **d** Schematic of the absorption process from the midgap state in the hybridized MoS_2_/Au layer. CBM, VBM and E_f_ are the abbreviation of conduction band minimum, valence band maximum and fermi level energy respectively. **e** F-P-like cavity formed in the MoS_2_/Au heterostructure. Light reflected from the top and bottom interfaces of MoS_2_ creates destructive interference

The interfacial condition of the MoS_2_/Au electrode depends heavily on the Au electrode fabrication method^31^. Metal atoms or clusters with high kinetic energy can collide with the MoS_2_ lattice at high speed during the metal deposition process and form covalent bonds with MoS_2_ or generate defects in the MoS_2_ lattice (Fig. 1a). This results in the formation of a hybridized interlayer at the MoS_2_/Au interface^35,36^. From an electrical point of view, the hybridization causes a shift in the Schottky barrier through Fermi-level pinning, which controls the contact resistance and carrier transport behaviour^31,36^. In addition, the hybridization layer at the interface influences optical properties such as absorbance^37^, which determines the number of carriers that can be generated in the material under light illumination. We studied the effect of various electrode fabrication methods on the absorption spectrum by fabricating three Au thin films on the same MoS_2_ flake using electrode transfer, thermal deposition, and sputter deposition, respectively (Fig. 1b). The samples being studied were fabricated using probe tip-assisted thin metal film transfer, and no adhesive polymer or liquid was used during the entire sample fabrication process. The detailed procedure is discussed in the Supplementary Information (Fig. S1). An ultraflat interface without hybridization can be expected with the Au transfer method. However, hybridization of MoS_2_ and Au might occur during two deposition methods. We observed that the absorption spectrum in the NIR range differed greatly across the three MoS_2_/Au heterostructures (Fig. 1c). However, we saw similar absorption at the A, B, and C exciton peaks. We observed absorption of 70% in the sub-bandgap wavelength with the sputter-fabricated heterostructure, which was considerably higher than that of their transferred (24%) or thermal-deposited (50%) counterparts. This indicated that the electrode fabrication method affected the absorption properties, and that the increased sub-bandgap absorption observed in the sputter-fabricated system might be attributable to the hybridization layer at the MoS_2_/Au interface. The F-P-like cavity formed in the MoS_2_/Au heterostructure plays a critical role in achieving high sub-bandgap absorption. Previous studies have reported that 2D materials with a high refractive index can form F-P-like cavities, which have a significant impact on the absorption spectrum and can increase overall absorption^33,38^. Detailed discussion on the F-P-like cavity will be provided in the explanation in Fig. 2e. Sputter deposition is a method that involves depositing metal films by bombarding a target with high-energy plasma ions. This collision imparts substantial kinetic energy to the deposition particles, causing them to sputter onto the surface of the samples with high deposition rate. As a result, sputter deposition achieves significantly higher energy levels compared to thermal evaporation. This distinction is evident from the deposition rate, with sputter deposition achieving a rate of 10 nm min^−1^, while thermal deposition only reaches 0.6 nm min^−1^. Need to note that the deposition rate is decisive for the sub-bandgap absorption. With the same deposition rate of 2.4 nm min^−1^ for both sputter and thermal deposition methods, the absorption spectra show a consistent trend (Fig. S2a), which implies that the collision of metal atoms with MoS_2_ is the primary driving factor behind the creation of midgap states at the electrode interface.

We verified our conjecture by using scanning transmission electron microscopy (STEM) to measure the cross-section of MoS_2_ with different Au thin film interfaces (Fig. 1d–f). The transferred Au (t-Au)/MoS_2_ heterostructure (Fig. 1d) exhibited a complete MoS_2_ lattice under the Au electrode, with a 6 ${\rm{\mathring{\rm A} }}$ distance between the Au and S atoms of MoS_2_. In contrast, the thermal- and sputter-deposited Au/MoS_2_ interfaces exhibited intimate contact between the Au and S atoms (Fig. 1e, f), indicating the presence of covalent bonds. There were a few blurry regions of the upper MoS_2_ lattice observed, which is the S vacancy, in Fig. 1e, f compared with Fig. 1d. This demonstrated the presence of interfacial defect states in the MoS_2_, as widely discussed in previous work showing that metal deposition can induce defects in the lattice and generate midgap states^35,39^. The extent of variation in the MoS_2_ lattice under the Au electrode in the sputter-deposited Au (d-Au) condition was relatively greater than that of its thermally deposited counterpart because whole-lattice distortion was observed in approximately four layers of MoS_2_. These STEM images demonstrated that the electrode fabrication method meaningfully affects the MoS_2_ lattice at the interface, thereby influencing the electronic and optical properties of the resulting device.

### Absorption spectrum modulation by controlling cavity resonance with MoS_2_ thickness

The hybridized layer increases NIR light absorption, and sub-bandgap light absorption is augmented if a hybridization layer is placed atop MoS_2_ as well. We fabricated such a sample by depositing an ultrathin layer of Au onto the MoS_2_/Au heterostructure. This ultrathin Au layer had a negligible effect on the absorption spectrum, as can be observed via optical microscopy and absorbance spectrum. (Fig. 2a, Fig. S2b, c). Absorption in the sub-bandgap wavelength increased to ~70% with the addition of this ultrathin Au layer (Fig. 2a). Furthermore, the absorption spectrum of a sample that had a similar configuration of layers but with a van der Waals interface rather than the hybridization interface of MoS_2_/Au exhibited a low absorption peak of 28% (Fig. S3), offering further evidence that the hybridization layer is critical for sub-bandgap absorption. Using atomic force microscopy (AFM), we measured the thickness of the ultrathin Au film to be 2.3 nm (Fig. S4) and confirmed the ultraflat surface of this film without nanoparticle formation, eliminating the possibility of plasmonic absorption. The depth of the hybridized layer for the sub-bandgap absorption was estimated to be 2 ~ 3 nm, which corresponds to 3 ~ 5 MoS_2_ layers_,_ based on the STEM image from previous paper^31^ and our result shown in Fig. 1f.

We further investigated the thickness-dependent absorption spectrum in the sub-bandgap regime. We constructed six MoS_2_ layers with different thicknesses ranging from 33 ~ 63 nm (Fig. S5), which demonstrated broad absorption in the NIR range, with an absorption peak redshift associated with increasing thickness of the MoS_2_ layer (Fig. 2b). We observed NIR absorption >60% for all six samples. The small drop in absorption seen with an increase in MoS_2_ thickness, notably from 70% at 24 nm to 60% at 63 nm, is likely attributable to the reduction in indirect band absorption. The peak wavelength shifted linearly in proportion to the MoS_2_ thickness (Fig. 2c), which is in good agreement with the F-P-cavity-like characteristics estimated using the transfer matrix method (Fig. 2c, red line) and widely studied in TMD materials^33,38,40^. This indicated that the sub-bandgap absorption peak could be modulated, and that this spectrum modulation was achieved by the MoS_2_ thickness. The high refractive index of MoS_2_ enabled the formation of an F–P-like cavity with a thickness of sub-100 nm. This anomalously strong NIR absorption can be explained through two mechanisms. In the first mechanism (Fig. 2d), sub-bandgap absorption occurs at the hybridized MoS_2_/Au interface^30^. This hybridized layer is highly n-type doped, which can be deduced from the transfer curve of the MoS_2_ field-effect transistor (FET) redshift after deposition of the ultrathin Au layer (Fig. S6). Hence, the midgap state was occupied with electrons, and these electrons can be excited to the conduction band of MoS_2_ by NIR light excitation. In the second mechanism, an F-P-like cavity was formed in the MoS_2_/Au heterostructure (Fig. 2e)^33^, maximizing the sub-bandgap absorption in the NIR wavelength range due to the occurrence of destructive interference. Hence, we could explain the comparable absorption of MoS_2_ on different electrode interfaces in the visible wavelength range in Fig. 1c. The formation of an F-P-like cavity within the MoS_2_ structure enhances absorption specifically at the wavelength of ~900 nm, while leaving the absorption in the visible range unaffected. Anomalously strong NIR light absorption in the broadband wavelength range was made possible within the hybridized MoS_2_/Au layer owing to these two mechanisms.

We propose that this electrode hybridization-enhanced NIR absorption augmentation is common for other 2D materials and metal configurations, because it originates from midgap state absorption in the TMD/metal hybridization layer and most metal deposition inevitably induces Fermi level pinning^41^ and generates midgap states in the interface. However, the levels of hybridization and absorption intensity are different, and this may depend upon the thin metal film absorption and midgap state formed at the interfacial layer. For example, we fabricated Cu and Pt thin films with a thickness of 2 nm via sputter deposition onto MoS_2_-Au and observed that both films exhibited absorption peaks in the NIR wavelength range but with different absorption values (Fig. S7). Further investigation is therefore needed to clarify the influence of different metal species on sub-bandgap absorption.

### Mechanism of photocurrent generation via sub-bandgap energy photons

**Fig. 3:**
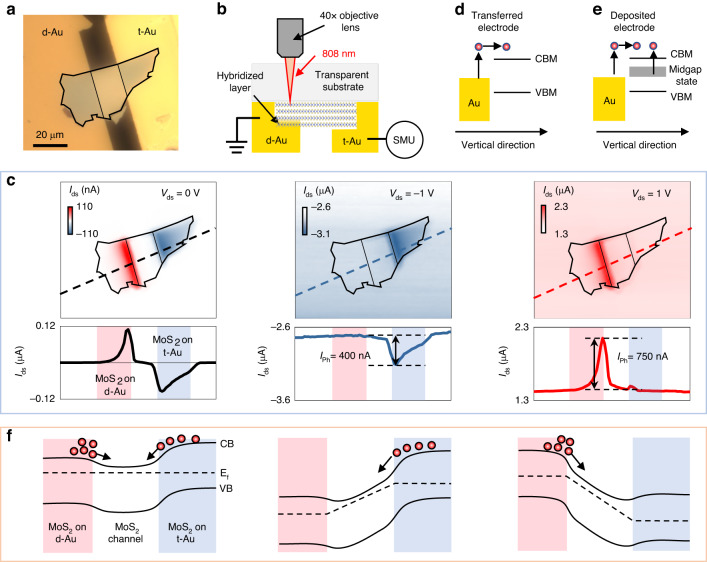
Photocurrent of MoS_2_ with sputter-deposited Au (d-Au) and transferred Au (t-Au) electrodes under the excitation of 808 nm. **a** Optical micrograph of the sample with the MoS_2_ thickness of 35 nm. **b** Schematic of the sample and photocurrent mapping system. SMU is the abbreviation of source meter unit. **c** Scanning images of photocurrent at bias voltages of 0, –1, and 1 V, respectively for left, middle and right panel (top). Bottom panels display the line profile of the dashed line in each image. Mapping shows the source-drain current (I_ds_) value acquired from laser beam scanning, and the measured photocurrent was derived by subtracting the background I_ds_ value from that of the region of interest. **d** Illustration of hot carrier generation/transfer in MoS_2_/t-Au. Electrons (red circle) are excited within the Au electrode by the 808 nm laser and transferred to the conduction band of MoS_2_. **e** Hot carrier excitation from the midgap state by the 808 nm laser in combination with hot electron transfer in MoS_2_/d-Au. **f** Band diagram of the device under 0, –1, and 1 V, respectively, for left, middle and right panel, illustrating the lateral electric field and the direction of photoexcited carriers contributing to the photocurrent

Strong NIR absorption was feasible in the MoS_2_ film through standard Au electrode fabrication methods. In this way, it is possible to generate carriers by NIR light excitation, which can be used for NIR photodetection. We fabricated a sample to explore the photocurrent generation mechanism at the MoS_2_/Au deposited electrode interface (Fig. 3a). The MoS_2_ layer was exfoliated onto a glass substrate, after which two Au electrodes were fabricated using sputter deposition and Au thinfilm transfer methods. We measured the optical microscopy and photocurrent mapping from the glass side to enable illumination of the MoS_2_ layer on top of the Au electrodes (Fig. 3b). Photocurrent mapping was performed by recording the source-drain current (I_ds_) during a focused 808 nm laser beam scan across the sample, such that the light-induced current difference (i.e., the photocurrent) could be observed from the mapping data. The resulting I-V curve of the sample exhibited an asymmetric shape (Fig. S8) due to the built-in junction formed in the lateral direction of the MoS_2_ layer and different Schottky junctions at the Au electrodes and MoS_2_ interface. We measured the photocurrent mapping at a bias voltage of 0 V, demonstrating that the photocurrent can only be generated in the MoS_2_ layer on top of the Au electrodes (Fig. 3c, left panel). Photocurrent was only generated at the electrode edge region on the d-Au side, although we observed a more uniform photocurrent in the overall MoS_2_ on the t-Au electrode side. The photocurrent also had an opposite direction for the two electrodes, indicating that the direction of electron flow was consistently from Au to MoS_2_ in both cases. We elucidate the mechanism of photocurrent generation using schematic illustrations in Fig. 3d, e. At the MoS_2_/t-Au interface, photocurrent can be occurred through hot electron transfer from Au to the MoS_2_ conduction band. This is facilitated by the excitation energy of 1.55 eV (800 nm), which exceeds the barrier at the MoS_2_/Au interface (Fig. 3d)^40^. However, at the MoS_2_/d-Au interface, both hot electron transfer and the midgap state absorption contribute to photocurrent generation. This results in a photocurrent that is twice as high as the MoS_2_/t-Au side (Fig. 3e). The electrons can be driven by the built-in potential formed in the MoS_2_/Au contact area and generate the photocurrent. It can be observed from the line profile of the left panel of Fig. 3c, that the maximum photocurrent with d-Au was slightly higher than that with t-Au. However, the d-Au electrode exhibits approximately threefold higher absorption (Fig. S9). This is because of the asymmetric built-in potential formed in the contact region owing to the asymmetric electrode; the junction in the MoS_2_/t-Au layer was higher than that in the MoS_2_/d-Au layer (Fig. 3f left panel). This results in decreased quantum efficiency of carriers generated in MoS_2_/d-Au even though greater carriers were generated by NIR light absorption.

We then applied a bias voltage of –1 and +1 V to the device to eliminate the effect of the built-in potential. The photocurrent mapping results are shown in middle and right panel of Fig. 3c. The photocurrent greatly increased by a factor of 10 after applying the bias voltage compared with that at 0 V, and only appeared on one side of the electrode. The negligible photocurrent under the channel region indicates that indirect band absorption-induced photocurrent in bulk MoS_2_ may be ruled out for wavelengths over 808 nm. The MoS_2_/d-Au layer generated a photocurrent with a maximum value of 750 nA when V_ds_ = 1 V, whereas a photocurrent of 400 nA was generated under a voltage bias of –1 V on the t-Au side. The magnitude of the electric field was similar under biases of –1 and +1 V that can be confirmed from the similar photocurrent, which are 6.6 μA and 6.5 μA for V_ds_ = −1 V and V_ds_ = 1 V respectively, under the 455 nm laser excitation (Fig. S10).

However, the sub-bandgap photocurrent generation area differed considerably. Photocurrent was generated in the entire region of MoS_2_ on top of Au, even at a 20 μm distance from the edge. Photocurrent was generated only at the edge area on the d-Au side, with a width of 7 μm. The maximum photocurrent was observed at the edge of the electrode and exponentially decreased with distance, which is attributed to the short electron lifetime at the hybridized interface. A two-fold higher photocurrent was observed on the d-Au side relative to the t-Au side due to the larger carrier density in MoS_2_ on d-Au, resulting from sub-bandgap absorption (middle and right panel of Fig. 3f; only carriers attributed to photocurrent are shown). In the early studies^14,17^, the hot electron transfer was inefficient in the longer-wavelength regime, and the photodetection properties worsened dramatically as the wavelength increased. In our case, however, SWIR detection by the deposited electrodes is relatively efficient because the electrode deposition induces mid gap state absorption and the subsequent photocarrier generation is unaffected by the wavelength in the sub-bandgap absorption.

### Photocurrent generation in MoS_2_ at telecommunication wavelength excitation

**Fig. 4:**
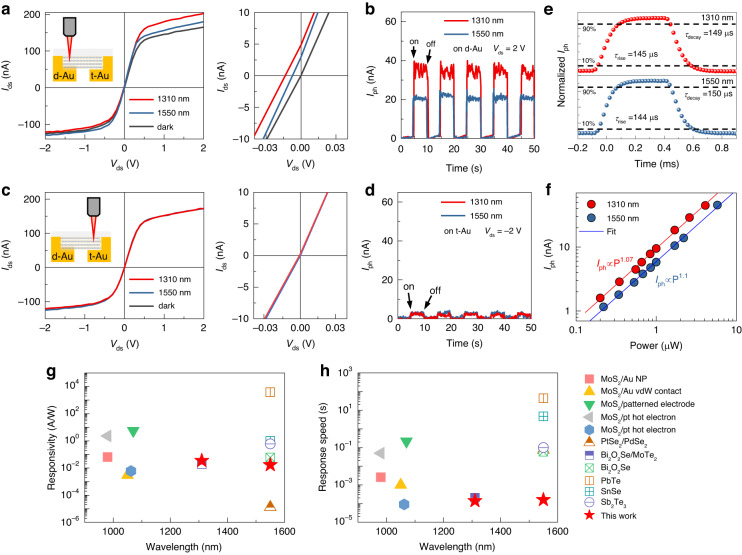
Telecommunication wavelength detection in MoS_2_/d-Au and t-Au electrodes. **a** I–V curve of MoS_2_/d-Au under dark conditions and illumination at 1310 and 1550 nm with a 1 μW laser focused with an 50X objective lens. Right panel is the magnified image of the small bias voltage to show the photocurrent under V_ds_ = 0 V. The different photocurrent at plus and minus voltage is due to the external bias voltage changing the electric field in MoS_2_ on d-Au, consistent with the results shown in Fig. 3. **b** Time-dependent photoresponse of MoS_2_/d-Au to 0.1 Hz square-wave light pulses with a 2 V bias voltage at 1310 and 1550 nm. **c** I–V curve of MoS_2_/t-Au under dark conditions and laser illumination at 1310 and 1550 nm. Right panel is the magnified image of the small bias voltage to show the negligible photocurrent under V_ds_ = 0 V. **d** Time-dependent photoresponse of MoS_2_/t-Au to 0.1 Hz square-wave light pulses with -2 V bias voltage at 1310 and 1550 nm. **e** Transient characteristics of the MoS_2_/d-Au device with 2 V bias voltage. **f** The incident power-dependent photocurrent of MoS_2_/d-Au under 2 V bias voltage. **g**, **h** The comparison of photoresponsivity and the response speed with the state of art 2D photodetectors^24,26,32,46–52^. Need to mention that only the papers that both reported the responsivity and response speed in NIR and SWIR wavelength range are considered here to ensure a more equitable comparison

We subsequently demonstrated that it is able to employ the MoS_2_/d-Au heterostructure to generate carriers in the conduction band of MoS_2_ using light wavelengths of 1310 and 1550 nm, thereby producing photocurrent. In this experiment, we used a thicker MoS_2_ of 80 nm thickness to realize high absorption in the telecom-band wavelength (Fig. S11). Since wavelengths of 1310 and 1550 nm cannot be detected by charge-coupled devices, we configured a custom optical setup to generate NIR illumination at a precisely targeted spot under the optical microscope (Fig. S12). We observed a notable increase in current in the plus bias voltage (V_ds_ from 0.5 to 2 V) when NIR light was projected onto the MoS_2_/d-Au (Fig. 4a). In contrast, we observed a negligible difference in the minus bias voltage (V_ds_ from −0.5 to −2 V), which is in close agreement with our previous photocurrent mapping results (Fig. 3c). The photocurrent under 0 V can also be generated if light is illuminated in the d-Au side (right panel of Fig. 4a). Spontaneous photocurrent of 5 nA and 3 nA for the 1310 nm and 1550 nm respectively was obtained. Photocurrents of 35 and 20 nA were generated under excitation at 1310 and 1550 nm under a bias voltage of 2 V (Fig. 4b). For MoS_2_/t-Au, in contrast, we observed a similar I–V curve whether the sample was excited at 1310 nm, 1550 nm, or under dark conditions (Fig. 4c), with a significantly lower photocurrent in the time-dependent response (Fig. 4d). The negligible photocurrent under the 0 V further confirms that the hybridized electrode is necessary for sub-bandgap photodetection (right panel of Fig. 4c). This result highlights that, under telecom-wavelength light excitation, the primary source of the sub-bandgap photocurrent at telecom-band wavelengths originates from the absorption induced by midgap states. Note that the contribution of hot electron transfer to photocurrent generation is negligible, in contrast to the scenario observed with 808 nm light (as depicted in Fig. 3d). We assessed photoresponse speeds for our MoS_2_/d-Au sample at 1310 and 1550 nm by switching the illumination on and off using a mechanical chopper at a frequency of 1 kHz (Fig. 4e). We observed a fast response speed in the range of 140 ~ 150 µs at both wavelengths. The light power-dependent photocurrent for this sample at both wavelengths is measured and plotted in Fig. 4f. In this plot, *I*_ph_ = *P*^*α*^, where *α* evaluate the gain induced photocurrent, was 1.07 and 1.1 for 1310 and 1550 nm, respectively, which indicated that photocurrent have linear dependence on the light power density and there was no extra gain during the photocurrent generation process^29^. Therefore, this system exhibits the potential for fast photoresponse. The linear photocurrent response as a function of the light power further rules out photothermoelectric effect^42^ as the mechanism of sub-band photocurrent. To demonstrate the universality of our method, we fabricated devices with WS_2_, MoSe_2_, WSe_2_, and an additional MoS_2_ device, and successfully demonstrate the NIR photodetection (Fig. S13 and S14). The statistical analysis of photoresponsivity of all MoS_2_ devices are displayed in Fig. S15.

We measured the time-dependent photoresponse of this system by illuminating the entire sample with wavelengths of 1310 and 1550 nm with the light power density of 11.6 mW cm^−2^ (Fig. S16). We saw a fast photoresponse (< 1 ms) followed by a slow response of several seconds in the photocurrent upon illumination at 1310 and 1550 nm. It can be deduced that the slow photoresponse was due to the bolometric effect^23^, whereas the fast response was due to sub-bandgap absorption (Fig. 4e and Fig. S17). The device responsivity is calculated with *R* *=* *I*_ph_*/P* where *I*_ph_ is the photocurrent and P is the power of incident light. For the photocurrent responsivity calculated with the fast response, which is generated from the sub-bandgap absorption, was 17 and 35 mA W^−1^ at 1550 and 1310 nm, respectively. Note that the bolometric effect induced photocurrent shown in the slow response regime is not included in the responsivity calculation. Our device demonstrates a detectivity of 8.5 × 10^7^ Jones at 1550 nm, which is slightly lower than that of other 2D materials. This is ascribed to the high dark current, which arises from the lack of additional engineering for low dark current, such as electrical gating. Furthermore, the external quantum efficiency (EQE) of our photodetector is 3.3% and 1.4% for 1310 nm and 1550 nm respectively. We also confirmed the reproducibility of our approach by making additional two devices with different device sizes, both presenting high photocurrent when light illuminated at d-Au side and negligible photocurrent at t-Au side as shown in Fig. S18. Further details on the performance of the devices, including the noise power density and reproducibility, can be found in Figs. 16–19.

## Discussion

We compared the performance of NIR photodetectors fabricated from TMD materials via different methods as shown in Fig. 4g, h. Our MoS_2_/d-Au system enabled NIR light detection at wavelengths up to 1550 nm, substantially outperforming other designs, with sub-bandgap detection in the wavelength range of 800 ~ 1250 nm (Fig. 4g). Our device also exhibited a far faster photoresponse (Fig. 4h), with the increase of 1–3 orders of magnitude. This exceptional device performance was due to the strong light absorption at the MoS_2_/Au interface and the low recombination rate at the MoS_2_ channel. Our approach offers a significant advantage over the hot electron photodetection technique, which has been extensively studied by the scientific community^28,32,43^. One of the major limitations of the hot electron approach is the rapid drop in responsivity at SWIR range. However, our approach overcomes this limitation and achieves high responsivity even in the SWIR range with the simple metal-semiconductor-metal (MSM) devices. Furthermore, the presence of a relatively high dark current poses a significant challenge for achieving highly efficient near-infrared (NIR) detection, especially for those small bandgap semiconductors. In comparison to materials such as PdSe_2_ and PtSe_2_, which exhibit elevated dark current levels of hundred μA^14^, our approach allows for the realization of low dark current. This is made possible by utilizing high band gap materials, which facilitate easier manipulation of majority carrier transport and effectively limit the dark current level. This makes our approach particularly useful for applications that require detection and imaging in the SWIR range, such as environmental monitoring, remote sensing, and medical diagnostics. Need to reiterate that the presence of the F-P-like cavity, formed by the TMD/metal electrode heterostructure, is crucial for enhancing the light absorption in the sub-bandgap region. This improved light absorption can boost photocurrent generation and the overall performance of the photodetector. Therefore, the design and optimization of the F-P-like cavity structure, particularly the selection of the appropriate thickness of TMD materials, are critical when aiming to achieve high-performance sub-bandgap photodetection.

In summary, interface hybridization via sputter deposition enabled our device to achieve sub-bandgap NIR light absorption in a thin MoS_2_ layer, with measured absorption as high as 60% around a wavelength of 1550 nm. The midgap state was generated in the hybridized layer and was highly n-doped, resulting in broad NIR absorption. Absorption was increased by the F-P-like cavity formed in the MoS_2_/Au heterostructure. In addition, the large refractive index of MoS_2_ enabled F-P resonance in the ultrathin MoS_2_. Therefore, the absorption wavelength could be modulated in a broad NIR wavelength range of 800 ~ 1600 nm by tuning the thickness of the MoS_2_ layer in the range of 20 ~ 80 nm. The strong sub-bandgap absorption was exploited to generate photocurrent, and the underlying photocurrent generation mechanism was studied in the MoS_2_/d-Au electrode. Telecom-band photodetection was feasible in our system, which presented high photoresponsivity of 17 and 35 mA W^−1^ under excitation at 1550 and 1310 nm, respectively, with a photoresponse speed of 140 ~ 150 μs. The optical properties of a material were engineered by the electrode fabrication method, and this study thus provides a practical approach for achieving sub-bandgap photodetection in 2D materials.

## Materials and methods

### Sample fabrication and characterization

Synthetic MoS_2_ (2D semiconductors) was exfoliated onto a PMMA spin-coated glass substrate from a bulk crystal using Nitto tape. Template striped ultraflat Au substrate^44^ was used to fabricate the F–P-like cavity. The probe tip-assisted metal transfer method^45^ was used to fabricate the mask onto the MoS_2_ surface (Fig. S1) for device fabrication. A 10 nm Ag/120 nm Au mask was used due to the low adhesion of Ag and 2D materials. Sputter deposited Au was deposited using radio frequency (RF) sputter (UHV Sputter-80P, A-TECH SYSTEM CO) in a high vacuum (10^−7^ torr) at a rate of 10 nm min^−1^. Thermal deposition of a 2 nm-thick layer of Au was performed in a vacuum chamber at 10^−7^ torr, with a deposition rate of 0.6 nm min^−1^, which was increased to 3 nm min^−1^ for additional thick Au layer deposition. The thickness of MoS_2_ was measured by an atomic force microscopy (AFM; NTEGRA Spectra DUO Max, NT-MDT). Cross-section STEM imaging of MoS_2_ and Au interface was performed with aberration-corrected STEM (JEOL ARM-200F).

### Absorption measurement

A home-built micro-absorption setup was used for the transmission and reflection measurements. White light (EQ-99X-FC LDLS, Energetiq Technology) was focused by the 50X NIR objective lens (M Plan Apo NIR 50X, Mitutoyo) and vertically irradiated onto the sample. Both transmitted and reflected light were collected by the objective lens and detected by a spectrometer (BLACK-Comet-SR for visible and DWARF-Star for NIR, Stellarnet). White light was focused to a 10 μm beam, which was smaller than the measured sample size. A protected silver mirror (PF10-03-P01, Thorlabs) was used for reference during the reflection measurement. The absorbance and the absorption of the sample were calculated from the transmission (T) and reflection (R) data, where absorbance = –log_10_T and absorption = 100 – R because transmission did not occur in the 120 nm Au film.

### Device measurement

Measurements were implemented under ambient conditions. A 2636B Source Measure Unit (Keithley) was used for the electrical measurement, and lasers with wavelengths of 455, 808, 1310, and 1550 nm (RGB lambda) were used for photocurrent mapping and response measurement. The time-dependent photocurrent response was measured by the light, which was mechanically blocked by the electrical flip stage (MFF101/M, Thorlabs). The photocurrent response speed at 1310 and 1550 nm was measured using an oscilloscope (DSO-X 2002A, Agilent Technologies), and a mechanical chopper (SR540, Stanford research) was used to cut the focused beam to switch the light on and off at a rate of ~100 μs. Photocurrent mapping was performed on the XPER RF (Nanobase). A two-leg optical fiber (RP21, Thorlabs) was used for the localized photocurrent measurement at 1310 and 1550 nm as shown in Fig. S12.

### Supplementary information

supplementary information
