# Supplementary material for: Engineering electrode interfaces for telecom-band photodetection in MoS2/Au heterostructures via sub-band light absorption
Source: Light Sci Appl. 2023 Nov 23;12:280. doi: 10.1038/s41377-023-01308-x (PMC10667329; doi:10.1038/s41377-023-01308-x)
Supplement: Supplementary file 1 — supplementary information [file 41377_2023_1308_MOESM1_ESM.docx]

**Supplementary information for**

Engineering electrode interfaces for telecom-band photodetection in MoS_2_/Au heterostructures via sub-band light absorption

Chengyun Hong^1,2^, Saejin Oh^1,2^, Vu Khac Dat^1,2^, Sangyeon Pak^3^, SeungNam Cha^4^, Kyung-Hun Ko^1,2^, Gyung-Min Choi^1,2^, Tony Low^5*^, Sang-Hyun Oh^5*^, and Ji-Hee Kim^1,2,6*^

^1^Department of Energy Science, Sungkyunkwan University, Suwon 16419, Republic of Korea

^2^Center for Integrated Nanostructure Physics (CINAP), Institute for Basic Science (IBS), Sungkyunkwan University, Suwon 16419, Republic of Korea

^3^School of Electronic and Electrical Engineering, Hongik University, Seoul 04066, Republic of Korea

^4^Department of Physics, Sungkyunkwan University, Suwon 16419, Republic of Korea

^5^Department of Electrical and Computer Engineering, University of Minnesota, Minneapolis, MN, 55455, USA

^6^Department of Physics, Pusan National University, Busan 46241, Republic of Korea

Correspondence: kimjihee@pusan.ac.kr, sang@umn.edu, tlow@umn.edu

**
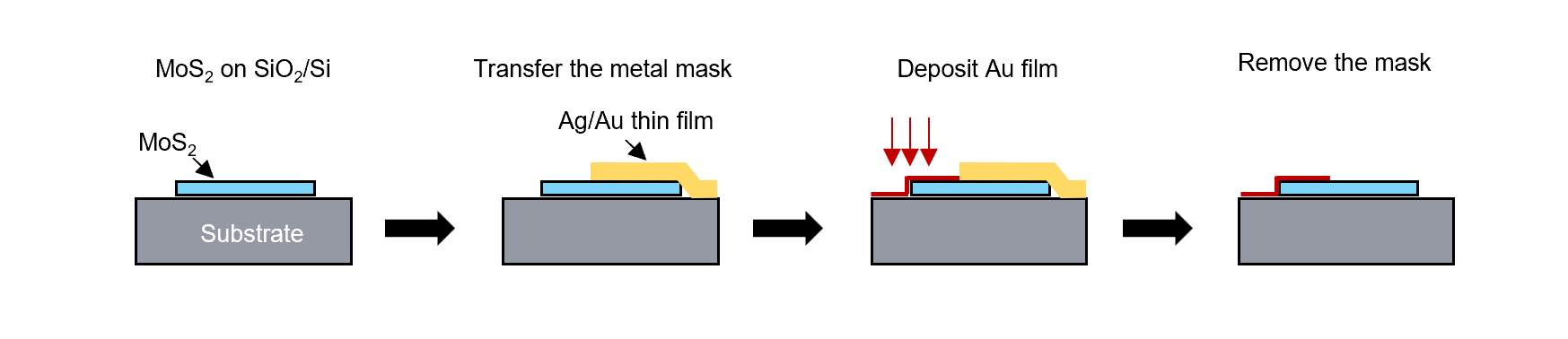
**

**Fig. S1** Schematic of localized metal thin film deposition. The probe tip-assisted metal transfer method was used here for mask fabrication. Specifically, the Ag/Au (10 nm/120 nm) film was deposited onto a SiO_2_/Si substrate using the thermal deposition method. The probe tip (ST-20-1, GGB industry) was used to cut the film to the desired size. The probe was pushed from one edge of the metal film and a hole was formed in the film to enable its removal from the substrate and transferred on top of the sample to function as a mask.^1^ After metal deposition, the mask could be easily removed by the probe tip. No impurities were present on the MoS_2_ surface due to the low adhesion of Ag and MoS_2_, as shown in Fig. S4.

**
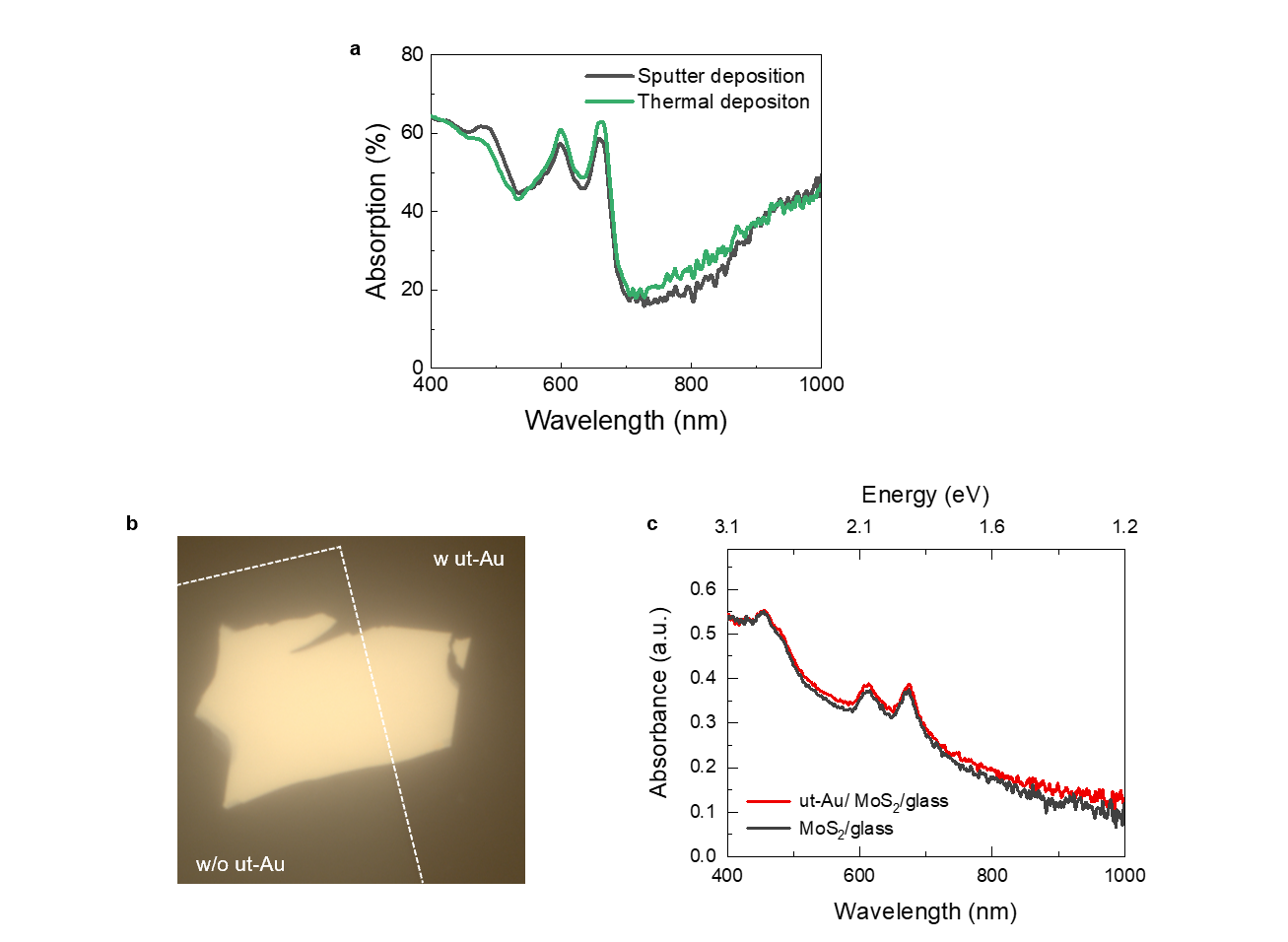
**

**Fig. S2 a** The absorption of MoS_2_ on sputter-deposited Au and thermal-deposited Au with the same deposition rate of 2.4 nm min^-1^. **b** Optical microscopy of MoS_2_ on a transparent substrate, with part of its surface deposited with an ultra-thin Au (ut-Au). The white dashed line is the edge of the ut-Au region. **c** Absorbance of the same MoS_2_ flake with and without ut-Au deposition.

**
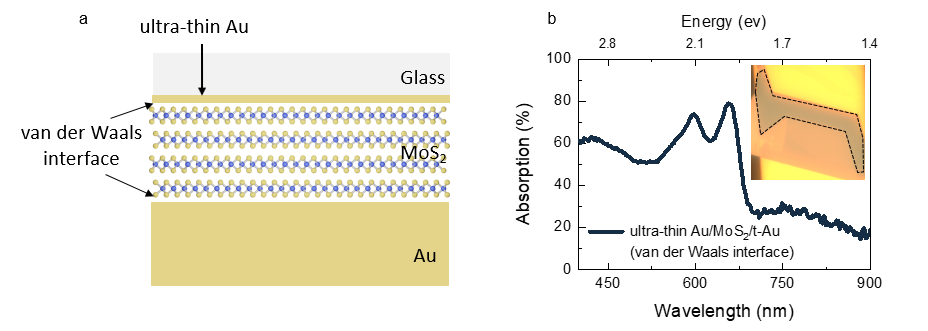
**

**Fig. S3 a** A sample consisting of the same configuration as shown in Fig. 2a, but where the interface between the ultra-thin Au layer and MoS_2_ is a van der Waals interface instead of a hybridized interface. **b** The absorption spectrum of this sample. Inset shows an optical micrograph of the sample, where the measured region is marked by a dark dashed line.

**
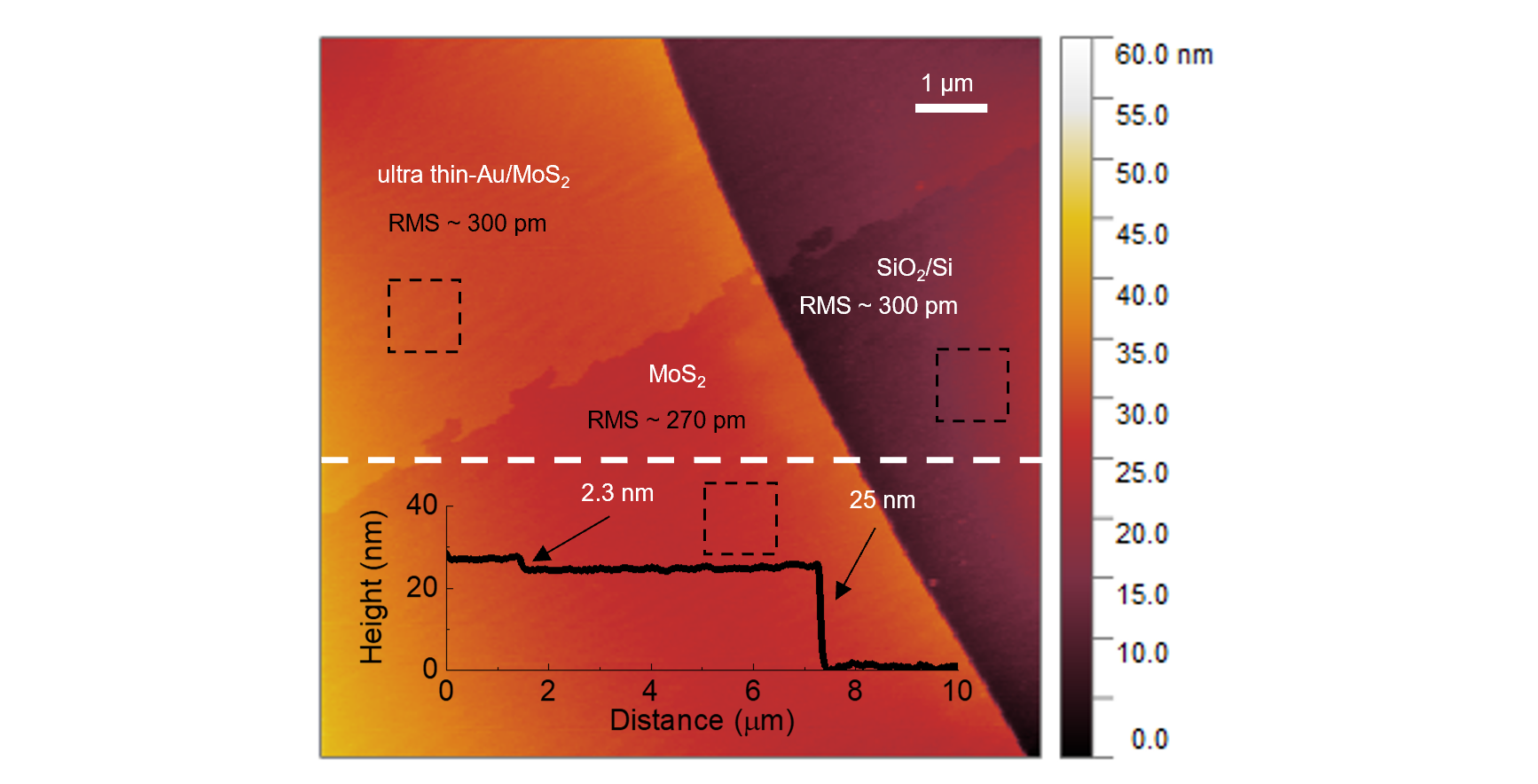
**

**Fig. S4** AFM measurement of ultra-thin Au deposited onto MoS_2_ on a SiO_2_/Si substrate, demonstrating the ultra-flat Au surface. Inset shows the 2.3 nm thickness of the ultra-thin Au film as measured from the line profile shown by the white dashed line. The regions of dashed black rectangles are used to get the roughness information.

**
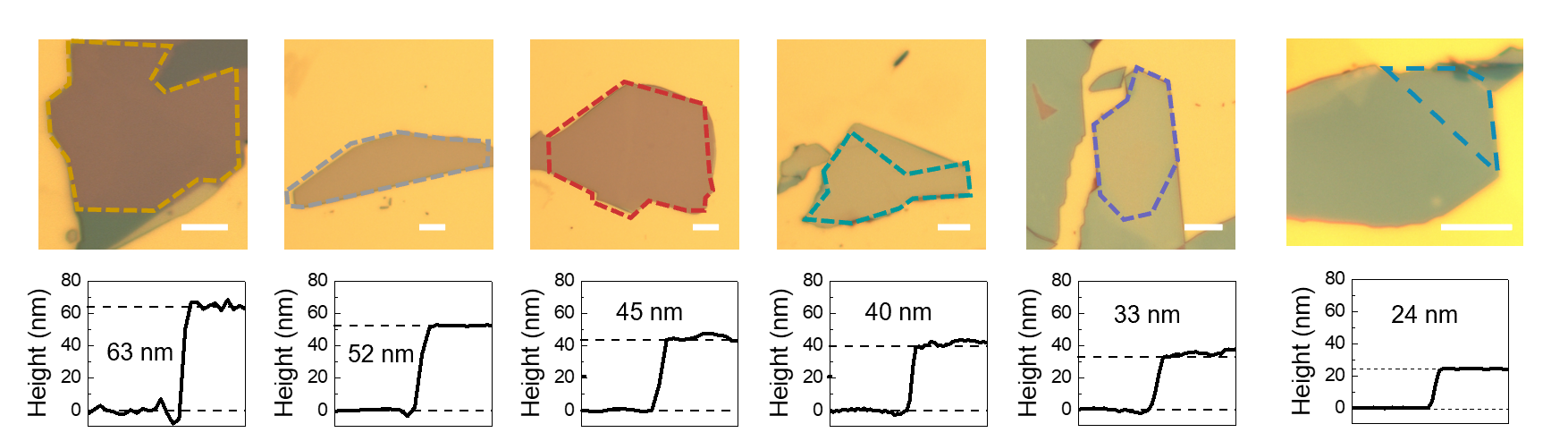
**

**Fig. S5** Optical micrographs (top) and AFM height profiles (bottom) for five samples with thickness ranging from 24–63 nm, corresponding to the Fig. 2a and Fig. 2b absorption data. The top MoS_2_ layer was hybridized via deposition of ultra-thin Au, as in Fig. 2a. scale bar: 10 μm.

**
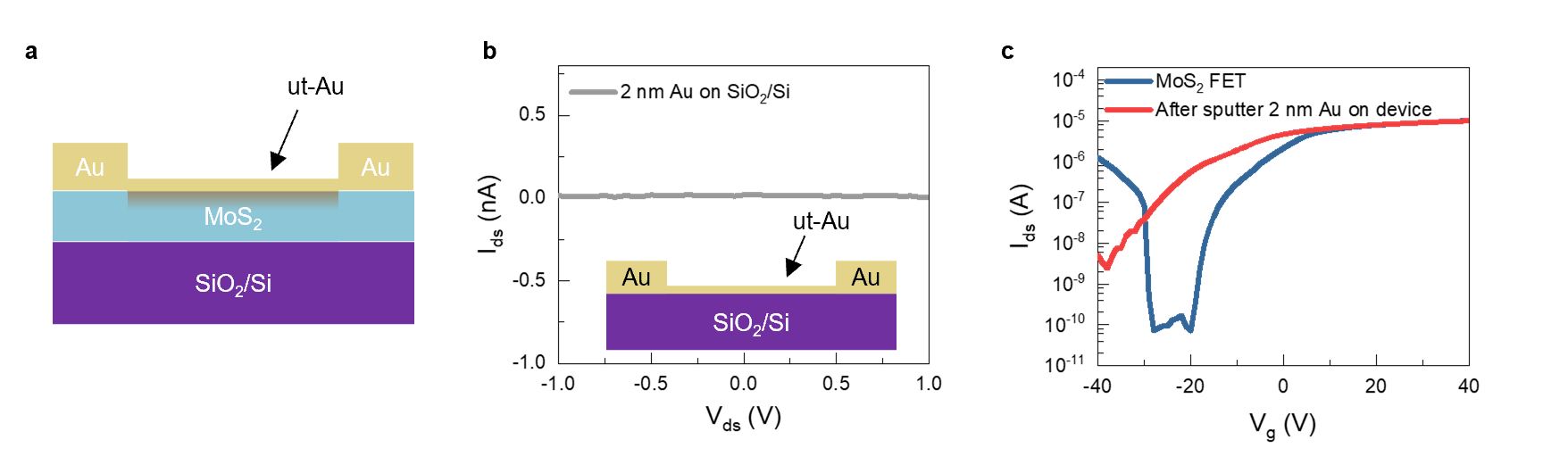
**

**Fig. S6 a** Schematic of a field effect transistor (FET) fabricated with MoS_2_ after ultra-thin Au deposition. **b** I-V curve of ultra-thin Au on the SiO_2_/Si substrate, showing negligible current flow. **c** The transfer curve before and after ultra-thin Au deposition on the MoS_2_ FET device, demonstrating strong n-doping after deposition.

**
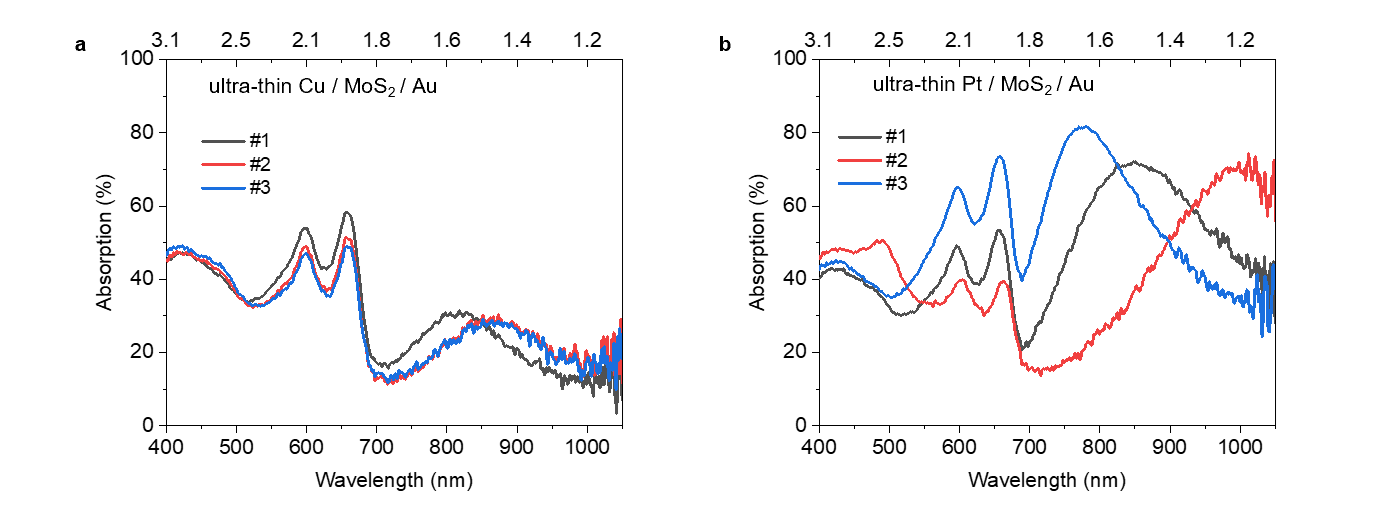
**

**Fig. S7** Absorption spectra of **a** ultra-thin Cu/MoS_2_/Au and **b** ultra-thin Pt/MoS_2_/Au with different MoS_2_.

**
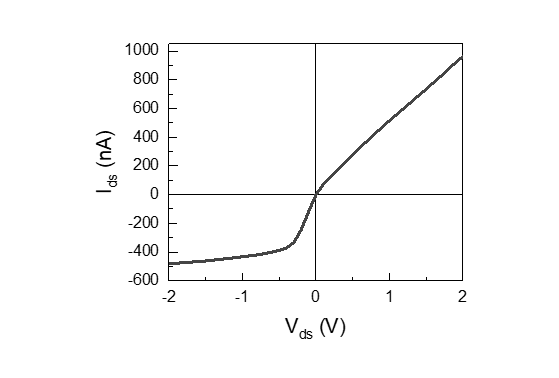
**

**Fig. S8** I–V curve of the sample shown in Fig. 3a demonstrates an asymmetric shape, which can be attributed to the built-in junctions in the MoS_2_ and different Schottky junctions in the two electrodes.

**
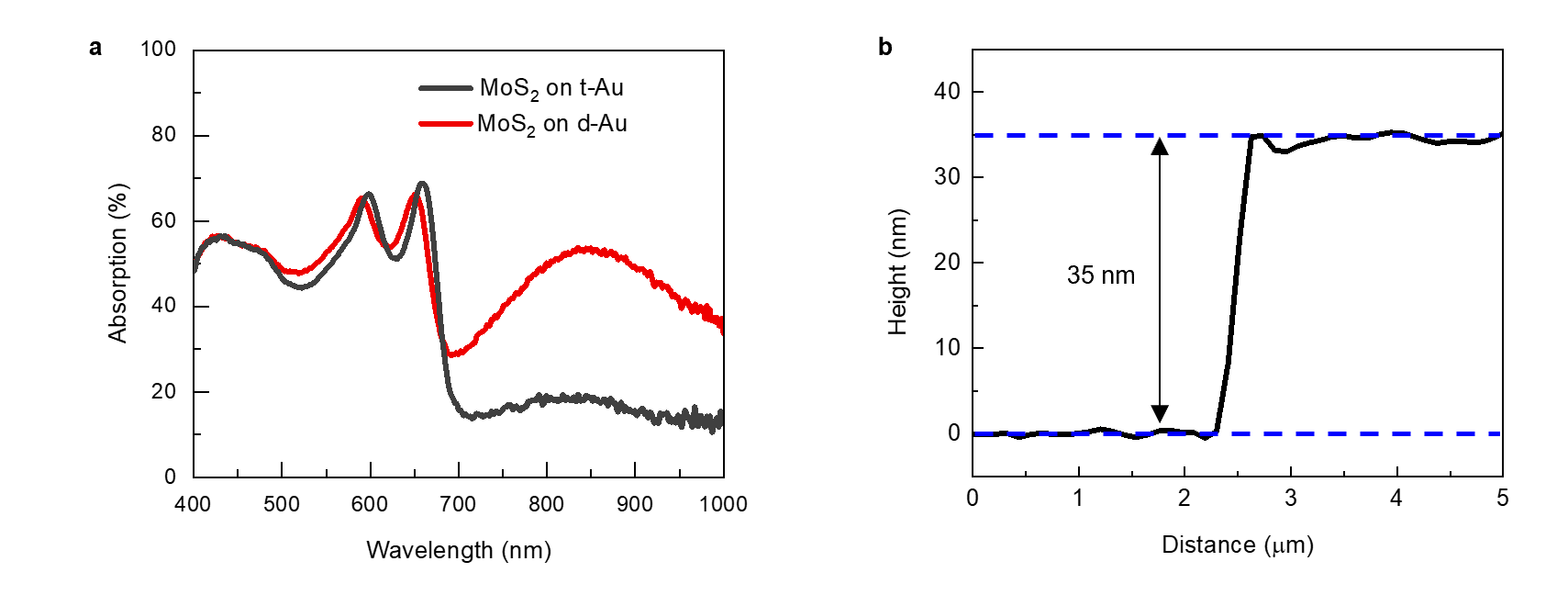
**

**Fig. S9 a** Absorption spectra of MoS_2_ on top of transferred Au (t-Au) and sputter-deposited Au (d-Au) in the device shown in Fig. 3a. **b** The thickness of the MoS_2_ consisted in the device in Fig. 3a.

**
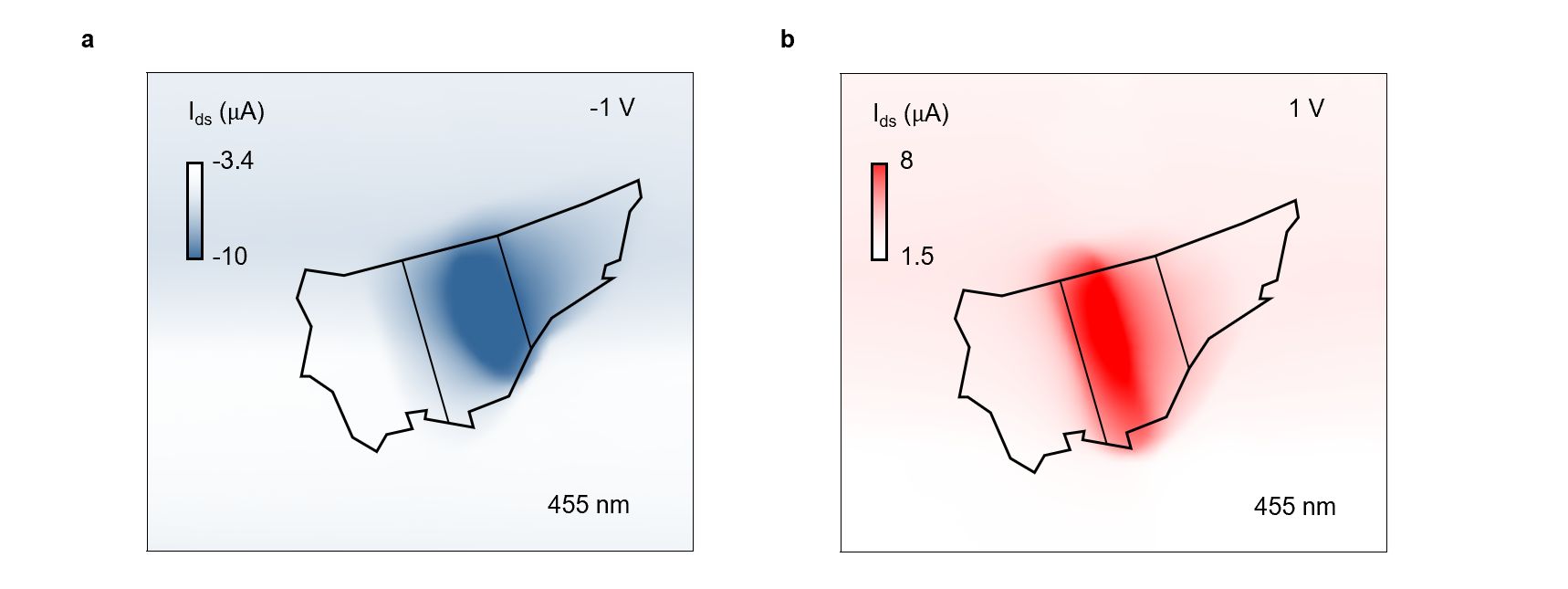
**

**Fig. S10** Photocurrent mapping data of the sample from Fig. 3a under a 455 nm laser with a bias voltage of **a**, -1 V and **b**, 1 V.

**
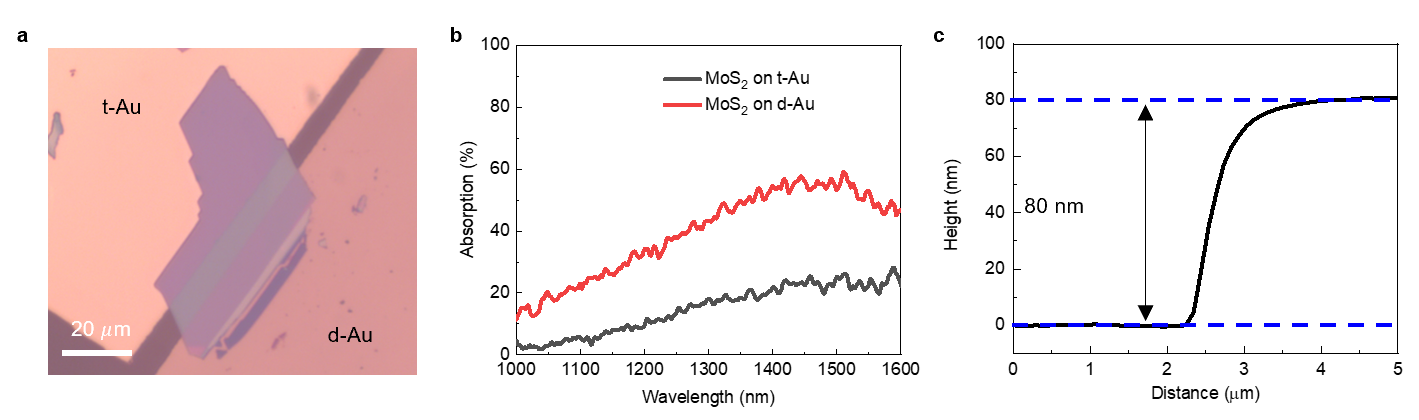
**

**Fig. S11 a** Optical micrograph of the sample measured in Fig. 4. **b** Absorption spectra from the MoS_2_ on t-Au and d-Au of the device. **c** The thickness of the MoS_2_ consisted in the device in Fig. S11a. Need to mention that the thickness of MoS_2_ for 1550 nm absorption is 80 nm, and it is slightly larger than the thickness obtained in Fig. 2c, which can be ascribed to the additional Glass/PMMA transparent substrate in the light path in our device.

**
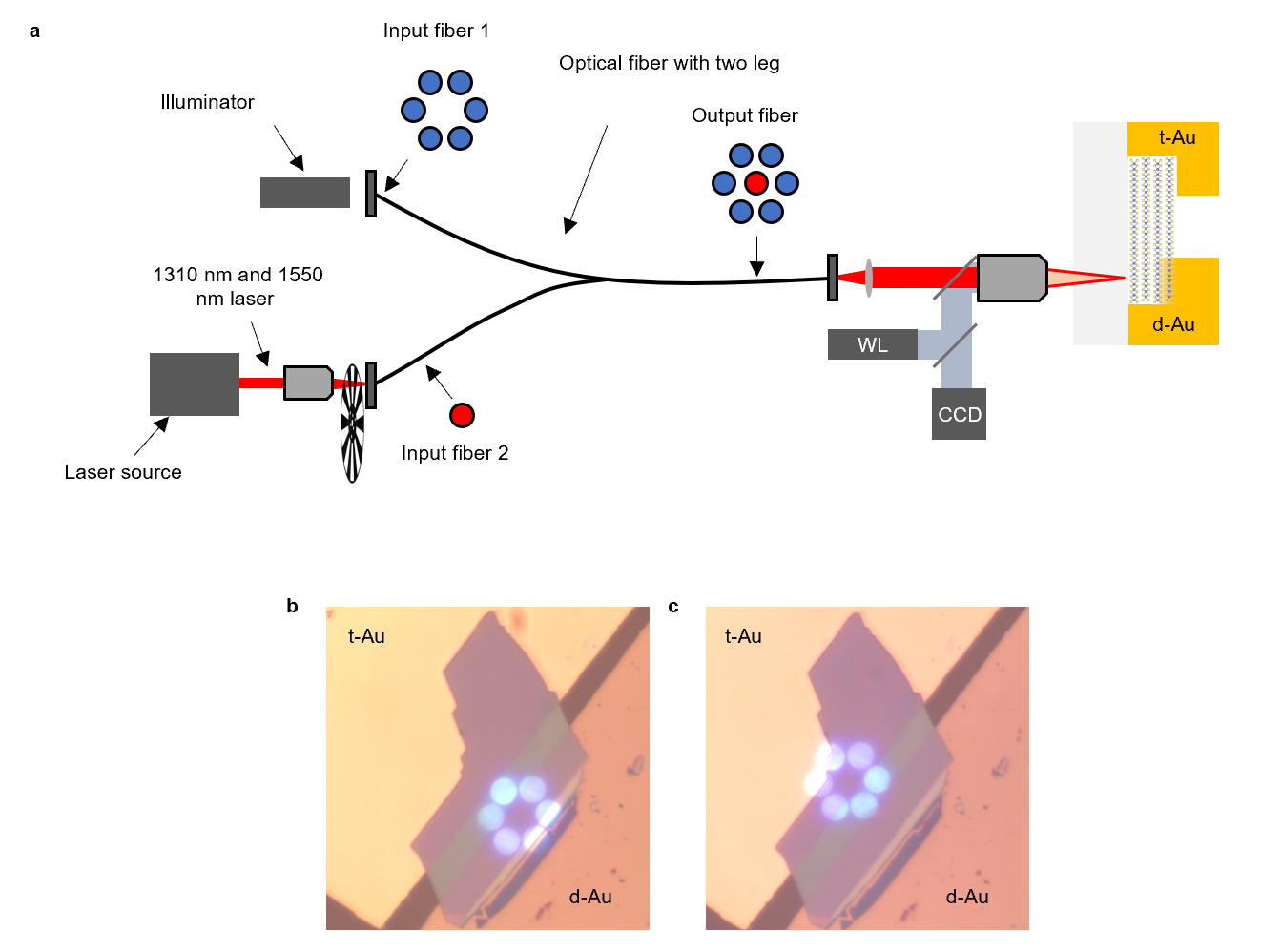
**

**Fig. S12 a** Experimental setup for localized 1310 nm and 1550 nm photocurrent measurement. An optical fiber with two legs was used to deliver the 1310 nm and 1550 nm illumination, which cannot be detected by the CCD camera on our optical microscope system. The six white light beam assists the position of NIR light. **b, c** Optical micrographs of the targeted NIR illumination on MoS_2_/d-Au and MoS_2_/t-Au, respectively.


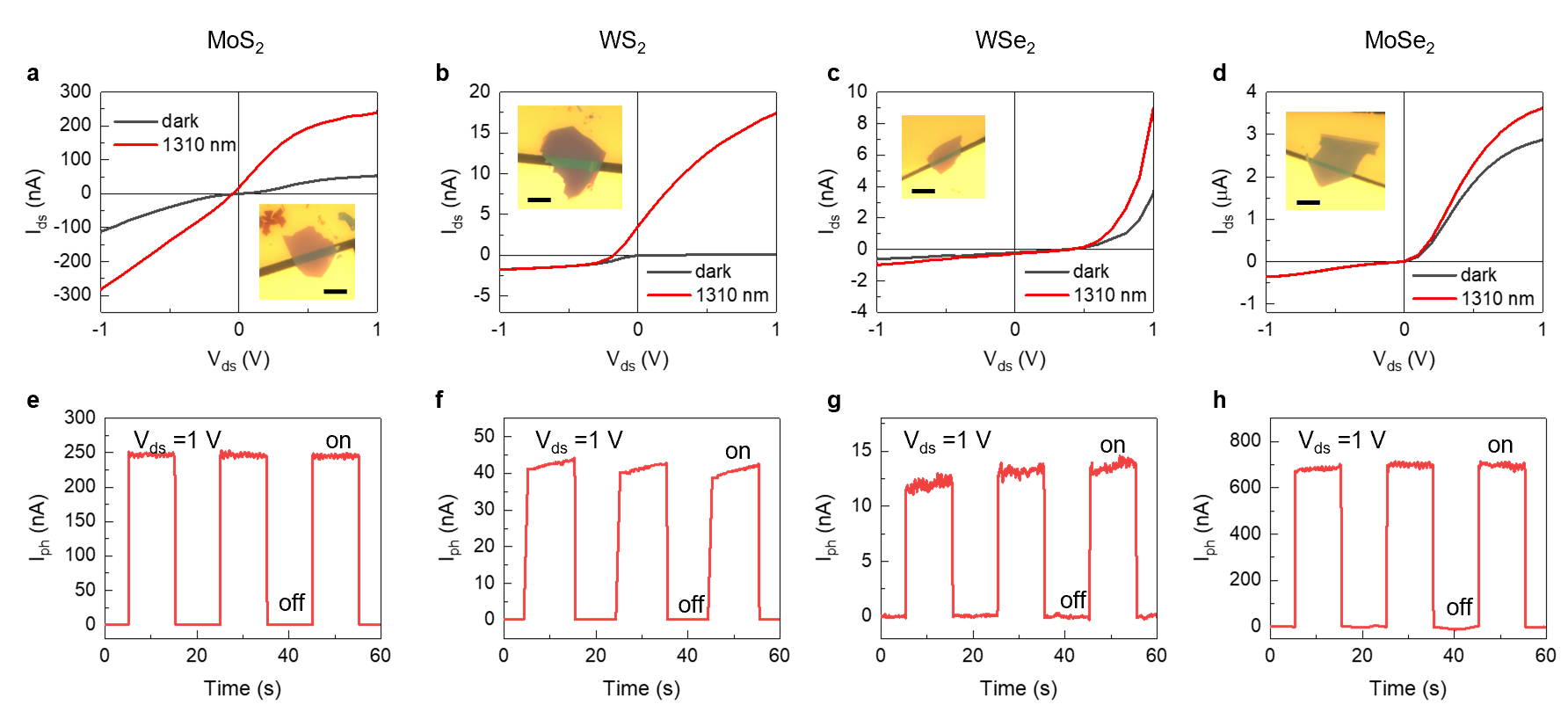


**Fig. S13** Photoresponse characterization of the device based on MoS_2_, WS_2_, WSe_2_, and MoSe_2_ under 1310 nm laser excitation. **a-d** The IV curve of the devices under dark and 1310 nm excitation with the power of 50 µW. The devices were fabricated with the same structure as Fig. 4, which consists of transferred Au and deposited Au serving as the electrodes, while the channel materials employed were MoS_2_, WS_2_, WSe_2_, and MoSe_2_. Inset: OM image of the device, scale bar: 20 µm. **e-h** The time-dependent photocurrent response under a bias voltage of 1V with the 1310 nm light on/off with 0.05Hz. We chose a thickness of ~ 80 nm for device fabrication.

All the devices exhibited sensitivity to both 1310 nm (Fig. S13) and 1550 nm (Fig. S14) laser excitation, although the photocurrent varied due to differences in the electric field conditions specific to each device. Notably, the MoSe_2_ device displayed the highest photocurrent, reaching 600 nA at 1310 nm and 150 nA at 1550 nm, with focused light powers of 50 µW and 21 µW, respectively. In contrast, the WS_2_-based device demonstrated a low dark current and considerable spontaneous photocurrent, along with the open circuit voltage of 176 mV and 145 mV under 1310 nm and 1550 nm illumination respectively. These characteristics are advantageous for achieving high detectivity and self-powered photodetection. Furthermore, we fabricated five MoS_2_ devices in our study and all devices demonstrated considerable photocurrent. The statistical analysis of photoresponsivity under 1550 nm illumination can be shown in Fig. S15, which presents good reproducibility.


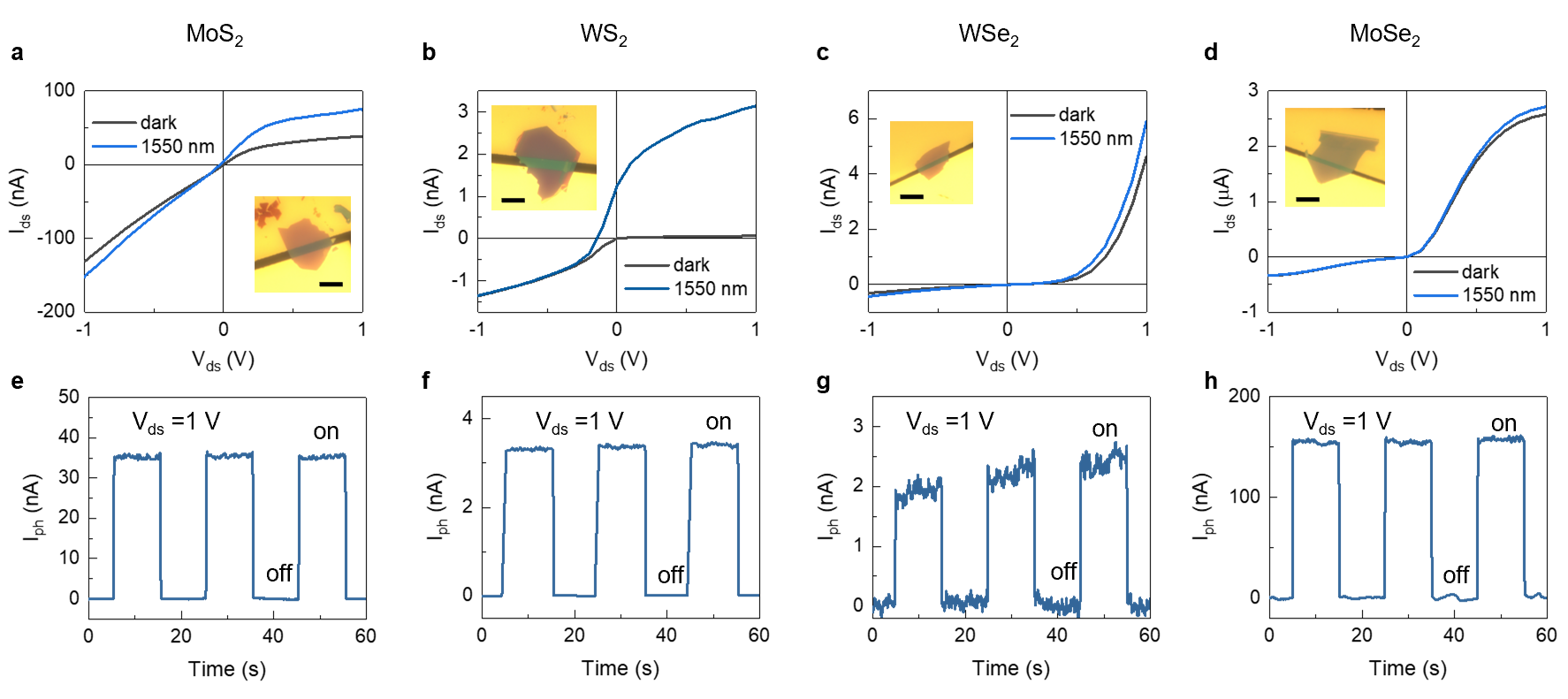


**Fig. S14** Photoresponse characterization of the device based on MoS_2_, WS_2_, WSe_2_, and MoSe_2_ under 1550 nm laser excitation. a-d The IV curve of the devices under dark and 1550 nm excitation with the power of 21 µW. The devices were fabricated with the same structure as Fig. 4, which consists of transferred Au and deposited Au serving as the electrodes, while the channel materials employed were MoS_2_, WS_2_, WSe_2_, and MoSe_2_. Inset: OM image of the device, scale bar: 20 µm. e-h The time-dependent photocurrent response under a bias voltage of 1V with the 1550 nm light on/off with 0.05Hz.


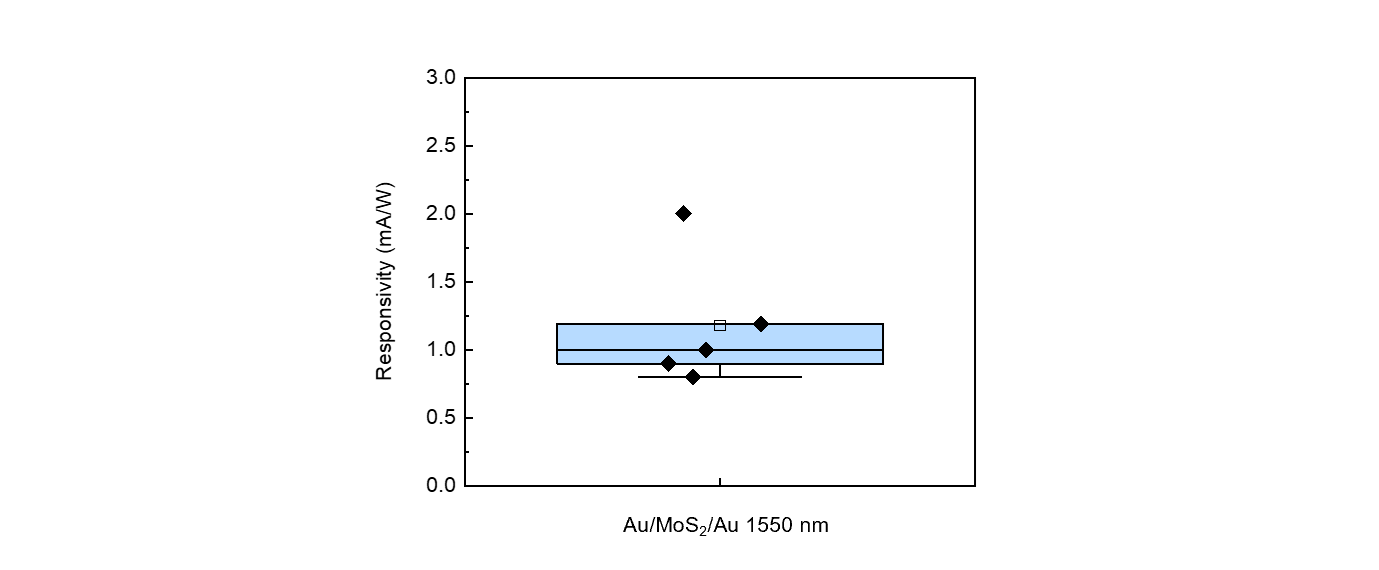


**Fig. S15** The statistical analysis of photoresponsivity of devices under 1550 nm illumination. We were able to observe sub-bandgap photoresponse in all the samples we fabricated. However, the photocurrent from different devices exhibited variations due to differences in device geometry. These deviations can be attributed to variations in device thickness, different channel lengths, and electrode contact area which might impact the built-in potential thus resulting in different photocarrier extraction efficiency.

**
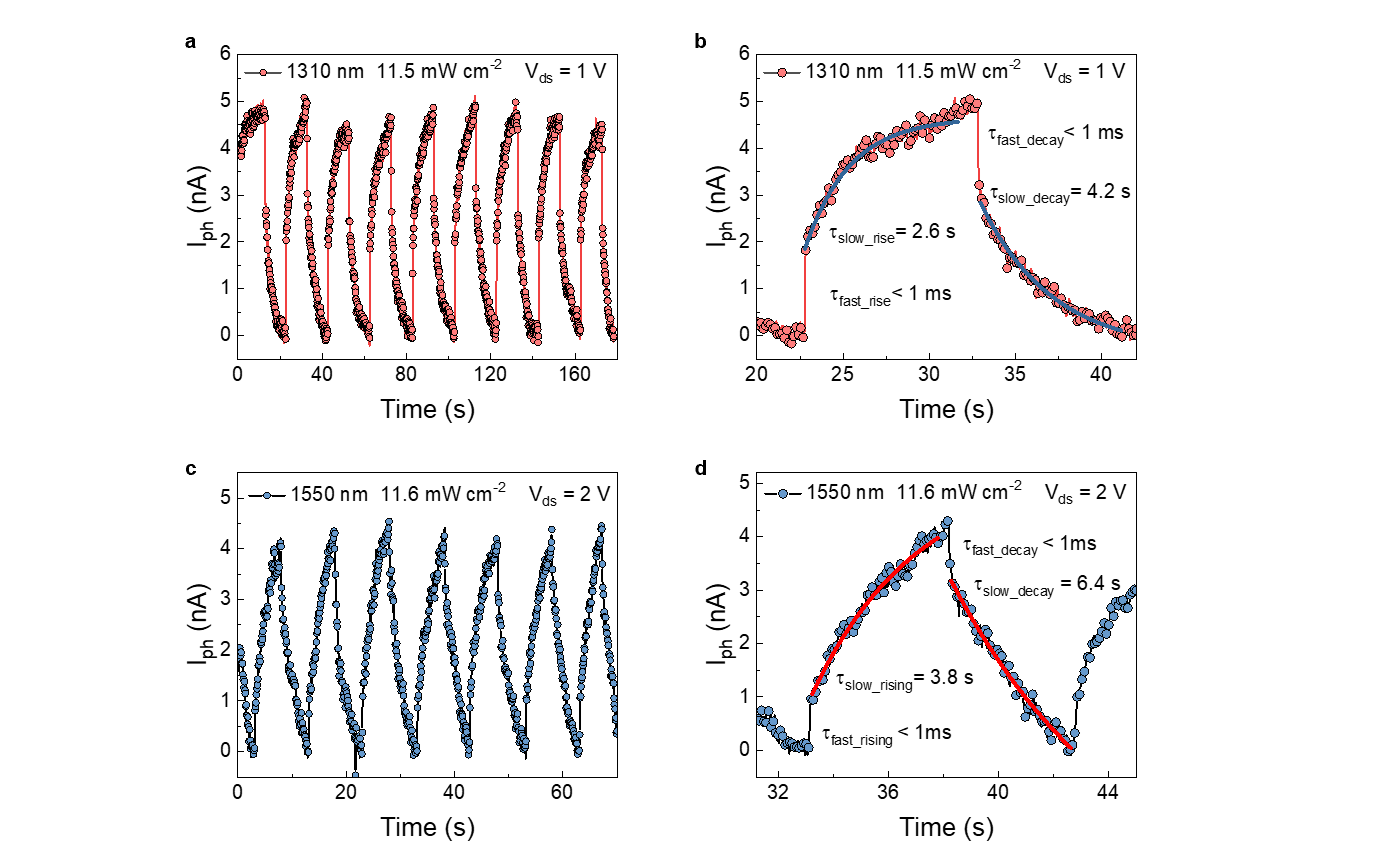
**

**Fig. S16 a** Time-dependent photocurrent of the sample from Fig. 4 exposed to 1310 nm illumination at 11.5 mW cm^-2^ power density. **b** Magnified waveform from panel **a**, demonstrating a fast response rate of < 1 ms and a slow response rate of several seconds. **c** Time-dependent photocurrent of the sample exposed to 1550 nm illumination at 11.6 mW cm^-2^ power density. **d** Magnified waveform from panel **c** demonstrating a fast response rate of < 1 ms and a slow response rate of several seconds. The detectivity *D** achieved in our device was 8.5*10^7^ Jones at 1550 nm calculated with the equation $D^{*}={{RA}^{1/2}}/{{(2eI_{\mathrm{dark}})}^{1/2}},$ where *R* is the responsivity, A is the active area, e is the unit electron charge, *I*_dark_ is the dark current. The relatively low detectivity in our device structure is primarily due to the high dark current, which is not reduced via special engineering as our primary focus was to demonstrate the electrode hybridization-induced photocurrent.

**
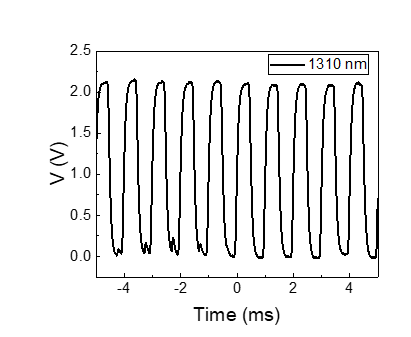
**

**Fig. S17** The raw data obtained from the oscilloscope corresponds to Fig. 4e.

_
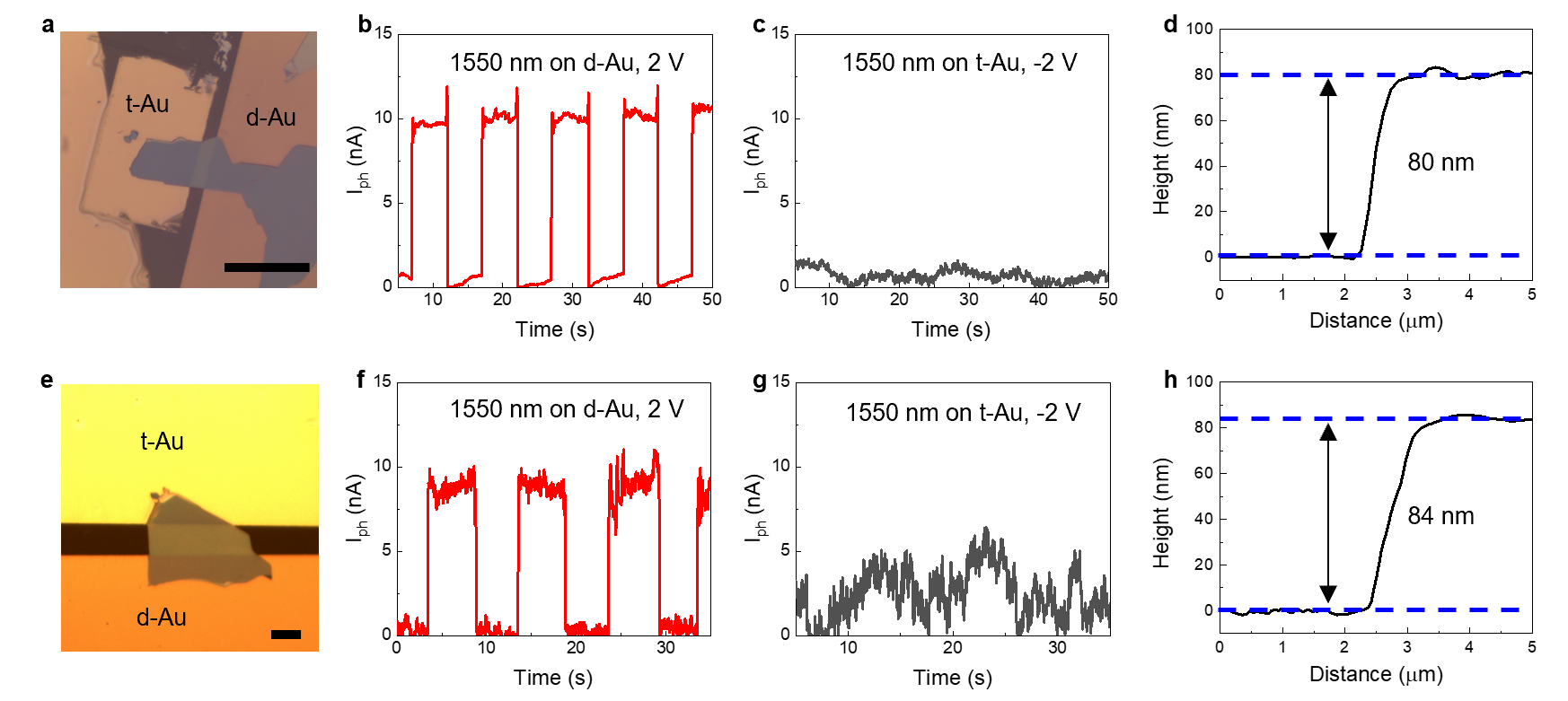
_

**Fig. S18 The photocurrent response under 1550 nm in two additional devices fabricated with different sizes of MoS_2_.** **a** The OM image of the device consisting of t-Au, d-Au, and 80 nm MoS_2_. **b, c** The time-dependent photocurrent under 1550 nm laser illumination on d-Au and t Au respectively. **d** the AFM thickness data of the sample in Fig. 14a. **e** The OM image of the device consisting of t-Au, d-Au, and 84 nm MoS_2_. **f**, **g** The time-dependent photocurrent under 1550 nm laser illumination on d-Au and t Au respectively. **h** the AFM thickness data of the sample in Fig. S14e. Scale bar: 20 µm. The reproducibility of our results can be confirmed with the additional two devices with different sizes as shown in Fig. S14, which exhibited high photocurrent under 1550 nm light in d-Au and negligible photocurrent under t-Au side. We assert that our approach is highly robust since interface hybridization occurs inevitably during the deposition process, except the special deposition method is adopted^2^, leading to an increase in photocurrent generation at the electrode interface and resulting in sub-bandgap photocurrent.

_
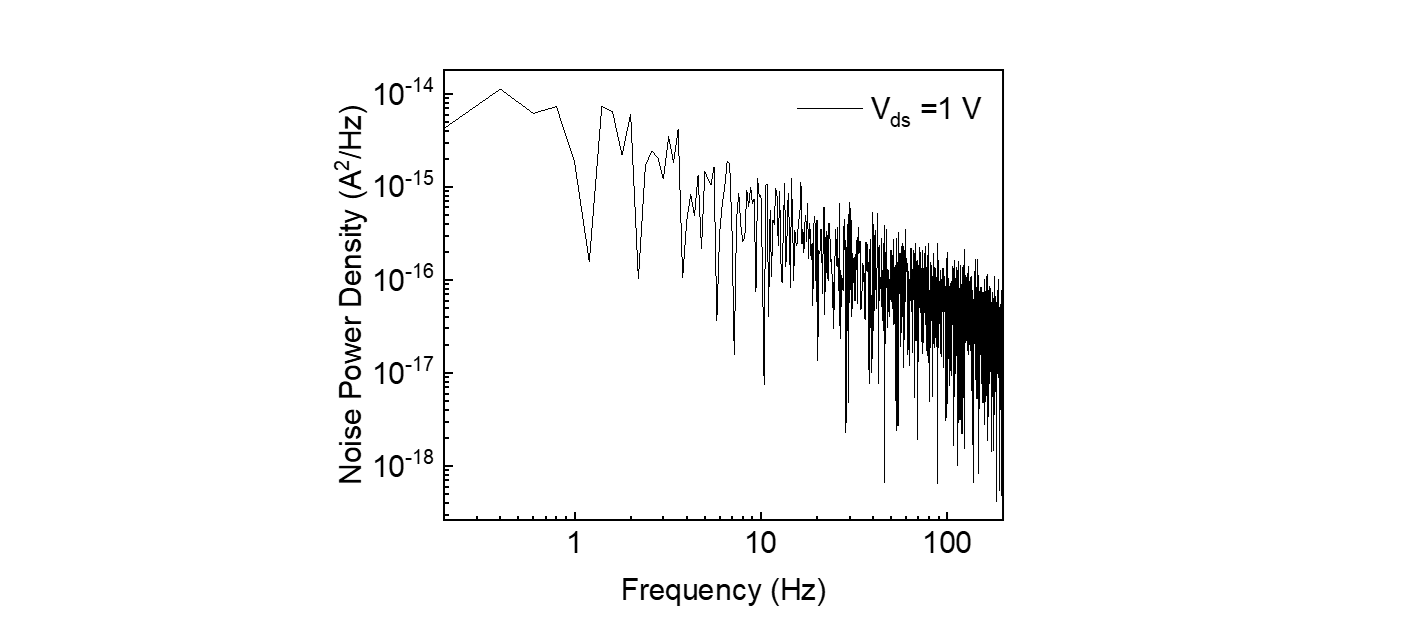
_

**Fig. S19** Noise equivalent power of the device. The relatively high dark current is due to the lack of additional engineering such as electrical doping in our MSM device.^3^

_
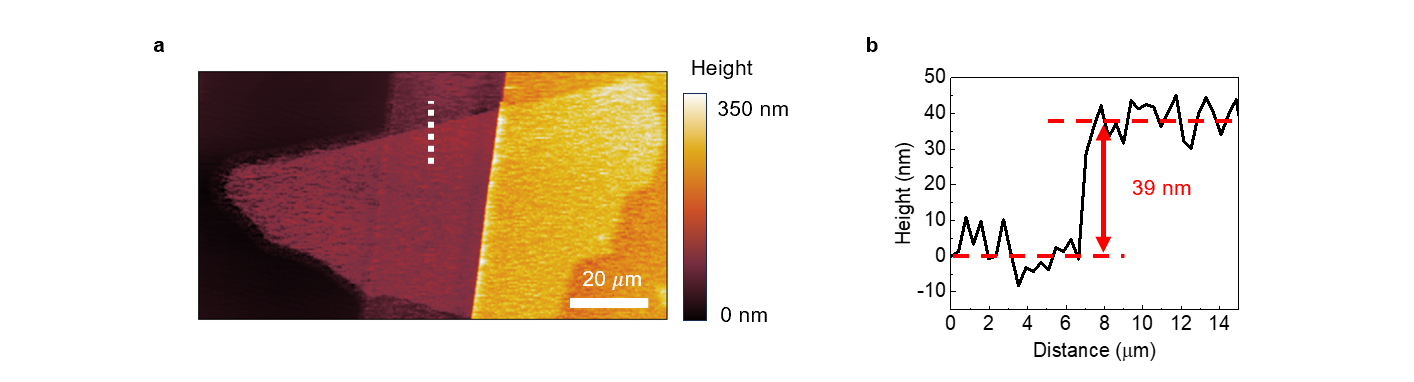
_

**Fig. S20** AFM result of the sample in Fig. 1c. **a** The AFM image and **b** height profile of the dashed line in Fig. S19a.

**Reference**

1 Liu, X. *et al.* Highly efficient broadband photodetectors based on lithography-free Au/Bi2O2Se/Au heterostructures. *Nanoscale* **11**, 20707-20714 (2019).

2 Wang, Y. *et al.* P-type electrical contacts for 2D transition-metal dichalcogenides. *Nature* **610**, 61-66 (2022).

3 Pak, S. *et al.* Electrode-Induced Self-Healed Monolayer MoS(2) for High Performance Transistors and Phototransistors. *Adv Mater* **33**, e2102091 (2021).
